# Enhancing sensitivity towards electrochemical miRNA detection using an affordable paper-based strategy

**DOI:** 10.1007/s00216-024-05406-6

**Published:** 2024-06-20

**Authors:** Wanda Cimmino, Ada Raucci, Sara Pia Grosso, Nicola Normanno, Stefano Cinti

**Affiliations:** 1 Department of Pharmacy, University of Naples “Federico II”, Via Domenico Montesano 49, 80131 Naples, Italy; 2 IRCCS Istituto Romagnolo per lo Studio dei Tumori “Dino Amadori”, Meldola, Italy; 3 Sbarro Institute for Cancer Research and Molecular Medicine, Center for Biotechnology, College of Science and Technology, Temple University, Philadelphia, PA 19122 USA

**Keywords:** Electroanalysis, Screen-printed electrodes, Paper-based, Preconcentration, miRNA, Cancer

## Abstract

In the era of liquid biopsy, microRNAs emerge as promising candidates for the early diagnosis and prognosis of cancer, offering valuable insights into the disease’s development. Among all the existing analytical approaches, even if traditional approaches such as the nucleic acid amplification ones have the advantages to be highly sensitive, they cannot be used at the point-of-care, while sensors might be poorly sensitive despite their portability. In order to improve the analytical performance of existing electroanalytical systems, we demonstrate how a simple chromatographic paper-based disk might be useful to rationally improve the sensitivity, depending on the number of preconcentration cycles. A paper-based electrochemical platform for miRNA detection has been developed by modifying a paper-based electrode with a methylene blue (MB)-modified single-stranded sequence (ssDNA) complementary to the chosen miRNA, namely miR-224 that is associated with lung cancer. A detection limit of ca. 0.6 nM has been obtained in spiked human serum samples. To further enhance the sensitivity, an external chromatographic wax-patterned paper-based disk has been adopted to preconcentrate the sample, and this has been demonstrated both in standard and in serum solutions. For each solution, three miR-224 levels have been preconcentrated, obtaining a satisfactory lowering detection limit of ca. 50 pM using a simple and sustainable procedure. This approach opens wide possibilities in the field of analytical and bioanalytical chemistry, being useful not only for electrochemistry but also for other architectures of detection and transduction.

**Supplementary Information:**

The online version contains supplementary material available at 10.1007/s00216-024-05406-6.

## Introduction

Cancer is the second leading cause of death globally, with 10 million deaths in 2020 [1], and the chances of survival are significantly increased if it is diagnosed and treated at an early stage [2]. Among the gold standard approaches for cancer management is tissue biopsy, but this is invasive and it is not suggested a repeated use [3]. As an alternative, liquid biopsy (LB) is currently revolutionizing the management of diseases, including cancer, by monitoring biomarkers circulating in biofluids [4]. Circulating biomarkers, such as nucleic acids, proteins, and extracellular vesicles, provide timely information on cancer diagnosis and progression [5, 6]. Liquid biopsy is promising because it offers the possibility of accessing bloodstream information reducing time, costs, and pain, often associated with tissue biopsies. Among the circulating biomarkers, miRNAs have been highlighted to be associated with different phases of cancer, from first appearance to metastases. These molecules can function as either oncogenes or tumor suppressors impacting a range of activities including cell growth programmed cell death and the spread of cancer cells, to parts of the body [7–9]. However, although miRNAs display a high potential as cancer biomarkers, their primary location within cells presents limitations, for their detection [10, 11]: traditional diagnostic methods like Northern Blotting [12], polymerase chain reaction (PCR) [13], microarrays [14], and next-generation sequencing (NGS) [15] are characterized by some analytical limitations, including the necessity of specialized equipment and trained personnel [16, 17]. To overcome these limitations, recent advancements in analytical techniques, such as colorimetric [18], fluorescence-based [19], electrochemiluminescence [20], and electrochemical techniques [21], have highlighted the easiness of application even complex matrices as the biological ones to detect traces of miRNA. The electrochemical ones are gaining high attention due to their sensitivity, cost-effectiveness, and inherent potential for miniaturization: these features pose electrochemical methods as wide adopted choice, especially towards diagnostics and miRNA sensing.

Various electrochemical systems have been reported for detecting miRNAs in biological fluids, by exploiting diverse approaches. In the work of Miglione et al. [22], an electrochemical biosensor was developed for the detection of miR-29a in serum. The platform was obtained by modifying screen-printed electrodes with gold nanoparticles and a thiolated capture probe labeled with methylene blue. The platform works according to the signal off principle: in the presence of the target, a less flexible probe-target hybrid is formed, resulting in a reduction of the methylene blue signal. In this work, the LOD obtained is 0.2 nM. A similar signal off-based platform was developed in the work of Yammouri et al. [23] for miR-21 detection. This biosensor is based on the immobilization of a thiolated methylene blue-labeled capture probe on the surface of a graphite pencil electrode modified with a carbon black/AuNPs nanohybrid, which allowed it to achieve a LOD of 1 fM. In this case, however, it is important to recognize that the process of preparing this sensor is time consuming. The electrode modification with carbon black alone takes more than an hour, followed by an overnight procedure for immobilization of the capture probe. An electrochemical biosensor for the detection of miR-492 was developed in the work of Moccia et al. [24]. In this study, paper-based screen-printed electrodes were used and a “signal on” strategy was adopted. The detection principle is based on the use of the redox mediator Ru(NH_3_)_6_^3^⁺, added to the working solution to monitor the formation of the PNA/miR-492 duplex. The redox mediator in this case accumulates on the working electrode due to electrostatic attraction with the negatively charged PNA/miR-492 hybrid. Consequently, in the presence of the target analyte, an increase in signal is observed, which resulted in a LOD of 6 nM. In the work of Zhu et al. [25], an electrochemical biosensor was developed to identify miR-21. This approach is based on the use of MoS2 nanosheets functionalized with thionine and gold nanoparticles. What makes this technique interesting is the use of thionine as a signal molecule to monitor DNA-miRNA hybridization. After successful hybridization of miR-21 with DNA, a DNA-miRNA duplex complex is formed hindering electron transfer, leading to a decrease in the electrochemical signal of thionine. This sensor is characterized by a LOD of 0.26 pM. However, the biosensor production time is time consuming; in fact, for the preparation of the sensor, this protocol requires the immobilization of the probe on the electrode surface for 16 h.

Although some methods have been developed, what should be noted is that the obtainment of very low detection limits is often attributed to the use of additional amplification procedures. In the work conducted by the group of Zuhang et al. [26], for example, enzymatic amplification using duplex-specific nuclease (DSN) was adopted. DSN is a nuclease specific for cleaving double-stranded DNA or DNA/RNA hybrid duplexes, but it is inactive against single-stranded DNA or RNA. The detection principle of this sensor is based on the use of a miRNA capture probe, which can form hybrids with miR-100. Then, DSN is added to cleave the probe-target hybrids created in the presence of miRNA in solution. The miRNAs thus released can then hybridize with other probes, and DSN is able to cleave these as well until all the DNA capture probes have been cleaved by the electrode. The cleaved capture probes release the electrode, thus allowing more interaction with the redox probes (K_3_Fe(CN)_6_/K_4_Fe(CN)_6_) to generate voltammetric signals. In this case, therefore, there is recycling of the target and an amplification of the signal that allows for low detection limit. In addition, among the amplification methods is catalytic hairpin assembly (CHA), a signal amplification strategy that occurs isothermally and without the use of enzymes. This method takes advantage of the DNA amplification cascade and strand displacement reaction to enable target recycling. Chao Zhao and colleagues [27] presented an electrochemical approach for simultaneous detection of miR-21 and miR-155. In their work, they designed four hairpin probes labeled with two electroactive groups, including ferrocene (Fc) and methylene blue (MB), to perform target-catalyzed CHA. In the presence of the target miRNA, CHA reactions were selectively activated between two hairpins labeled with either Fc or MB, producing the products CHA 21 and CHA 155 depending on the miRNA present in solution. Subsequently, these products were captured by PNA-21 and PNA-155 probes, bringing the Fc and MB molecules close to the electrode surface, and generating enhanced electrochemical signals, measured by square wave voltammetry. This sensor has a limit of detection of 2.36 fM for miR-21 and 10.56 fM for miR-155. Molecular amplification is another strategy developed to overcome current challenges in miRNA analysis and has shown high amplification efficiency for oligonucleotides. Ling-Li Zhao [28] reported an electrochemical method for miR-let-7a detection based on the specific binding of G-triplex (G3) with methylene blue. The G3-EXPAR method uses a novel molecular amplification strategy divided into two steps: a linear amplification step and an exponential amplification step. This process converts the target miRNA into G3 molecules, which, after being labeled with MB, are detected by the sensor. This process resulted in a LOD of 0.45 fM. The introduction of amplification strategies appears to have improved the sensitivity in miRNA detection, allowing for the detection of very low levels, even in the order of attomolar. However, many of these strategies require the use of enzymes or other biomaterials, which can increase the costs and complexity of the analysis. Additionally, long incubation times are often required, limiting the application of these methods as routine point-of-care (POC) devices. To this regard, sample treatments as the adoption of preconcentration strategies have shown to improve the sensitivity of reported approaches. Ultracentrifugation [29] and filtration [30] are considered the gold standard. The ultracentrifugation method separates biomolecules based on their density but is expensive and requires expertise, analysis time, and appropriate equipment. Alternatively, the filtration method separates biomolecules according to their size but has various limitations such as low selectivity and low yield, and low selectivity. In addition, the sample can be damaged during the process due to non-specific binding to the filter. Highly efficient and selective preconcentration techniques with simple operation applied to analytical methods are needed to detect the sample at low concentration. In addition to these methods, new approaches for miRNA preconcentration have been developed, including the use of magnetic nanoparticles or the use of electrokinetic. For instance, Ustuner et al. developed an assay to improve the accuracy and reliability of sensors in detecting microRNAs. The method uses magnetic spheres to capture target microRNAs from solution, allowing preconcentration of the sample prior to electrochemical detection [31]. The magnetic beads were modified with streptavidin and LNA probes specific for the target miRNA; the nanoparticles thus functionalized are placed in contact with the solution to be analyzed. After that, denaturation of the LNA-miRNA hybrid to release the target from the nanoparticles occurred in a 1 M NaOH solution. Their proposed method demonstrated the ability to detect miRNA concentrations with a LOD of 0.38 fM. However, it has several disadvantages that limit its applicability, especially in point-of-care diagnostic settings. First, the preconcentration step requires long incubation of the target with the magnetic nanoparticles for 30 min. Next, the particles must be washed repeatedly in PBS to remove any impurities. Finally, denaturation of the LNA-miRNA hybrid is required, which further complicates the procedure. Together, these steps make the procedure time consuming and complex, with a number of steps that make it impractical for implementation in point-of-care diagnostics. In addition, the presence of specific steps that are difficult to perform could limit the accessibility and usability of the platform, making it user-unfriendly. Preconcentration with ion concentration polarization (ICP) is considered an excellent candidate for preconcentration of biological molecules because ICP can rapidly concentrate and separate charged particles by a simple electrical operation without the use of chemicals. For example, a three-dimensional paper-based microfluidic analytical device (3D-μP2) was developed in the work of Yannan et al. The device uses the ICP to preconcentrate the analyte in solution, which is then detected with a colorimetric assay. With this approach, the LOD of the colorimetric assay was increased by 3 orders of magnitude compared with the unpaired assay [32]. Despite the good results obtained from these approaches, some drawbacks are observed: the use of magnetic beads as mentioned has the disadvantage of lengthening the assay time, while devices using electrokinetic are complex to design and build, especially for more advanced applications. Managing interfaces between regions of high and low ion concentration requires precise design and careful control of parameters. These drawbacks make these approaches not entirely suitable for user-friendly and decentralized applications, with one main problem being the increased complexity of the entire analytical procedures. Herein, to overcome current limitations in terms of costs, customized receptors, time duration, and summative washing steps, we propose a simple and general approach for preconcentration that can be applied to a large example of analytical methods. In particular, a miniaturized and wax-printed chromatographic paper-based disk has been used to preconcentrate miRNA present in both standard and human serum solutions. No additional reagents nor complex procedures have been required to preconcentrate miRNAs up to tenfold, down the picomolar level. As a case of study, an electrochemical paper-based sensor customized with gold nanoparticles and a DNA recognition probe has been considered for the detection of miR-224, which is associated with lung cancer prognosis. The adoption of paper-based preconcentration leads to many advantages, covering various features such as the material’s sustainability itself, the customization with wax printer, the easiness to be applied everywhere, and the low cost (less than a Euro cent). It represents a frugal example on how the use of available materials, i.e., porous paper, is effective in improving the sensitivity without affecting the costs of measurements, only adding 10 min to the whole process.

## Experimental section

### (Bio)reagents, chemicals, and materials

Sodium chloride (NaCl), chloroauric acid (HAuCl_4_), sodium borohydride, sodium citrate, PBS tablets (140 mM NaCl, 10 mM phosphate buffer, 3 mM KCl), 6-mercapto-1-hexanol (MCH, C_6_H_14_OS), tris(2-carboxyethyl) phosphine (TCEP; C_9_H_15_O_6_P), and human serum were purchased from Sigma-Aldrich (St. Louis, MO, USA). The MB-DNA probe selective to miR-224 (5′-Thiol-C6-CTA AAC GGA CCA CTA GTG ACT TGA-methylene blue-3′), the target sequence (5′-uca agu cac uag ugg uuc cgu uua g-3′), and the control sequences used in selectivity study were purchased from Metabion GmbH (Steinkirchen, Germany). Paper-based graphite SPEs were in-house produced using office paper [33]. The three-electrode design was manually screen printed, as previously described [33, 34]. The conductive silver ink was purchased from Loctite (Italy) and the carbon ink was purchased from Sun Chemical (USA). Gold nanoparticles (AuNPs) have been synthetized as described in a previous work [22]. All the electrochemical measurements were carried out using a portable potentiostat PalmSens 4 (PalmSens, Netherlands) equipped with a multi-8 reader and interfaced to a laptop using PSTrace5.9. Square wave voltammetry was recorded in a potential range 0.00 to − 0.6 V with an amplitude of 0.01 V and a frequency of 50 Hz. The disks used for sample preconcentration were manufactured by hand.

### Screen-printing and AuNPs synthesis

First, the glassware and magnetic rod used in this synthesis were cleaned in aqua regia (HCl/HNO_3_ 3:1 (v/v)), rinsed in distilled water and then cleaned with piranha solution (H_2_SO_4_/H_2_O_2_ 7:3 (v/v)), and rinsed again with distilled water before use. Next, gold nanoparticles (AuNPs) were obtained in a laboratory ball at room temperature (RT) by mixing 9 mL of distilled water with 1 mL of HAuCl_4_ at 0.01 g/mL and 2 mL of sodium citrate at 0.01 g/mL. Next, 500 mL of 20 mM sodium borohydride was added drop by drop. The solution was left under stirring and in the dark overnight. The dispersion of AuNPs was then stored at 4 °C. Office paper electrodes were used for this study to produce the biosensor. To produce the screen-printed electrodes, a ColorQube 8580 office printer from Xerox (USA) was used to print a specific hydrophobic pattern on the office paper. The design of hydrophobic barriers was created through a drawing software, specifically Adobe Illustrator. Subsequently, the paper with the wax-printed pattern was subjected to a 1-min heat treatment at 100 °C in an oven. This heating process induced the melting of the printed wax, which then permeated the substrate, forming a hydrophobic barrier. On this wax layer, the electrodes were printed using screen-printing technology. Screen-printing is one of the thick-film techniques that is widely used for mass production. The inks are printed onto a substrate directly through a mask-net with a designed pattern. The electrical contacts and the reference electrode were printed with a silver ink and the counter, and the working electrode was printed with a carbon ink. The diameter of the working electrode is 4 mm, while the electrochemical strips measure about 2.5 cm in height by 1 cm in width.

### Preparation of the office paper-based strip for miRNA detection

To construct the biosensor, the surface of the working electrode was modified with 4 mL of AuNPs via drop casting. The sequence of anti-miR-224 was customized by introducing a thiol group at the 5′ end. This modification facilitates the immobilization of the DNA probe on the electrode surface through Au–S bond. To anchor the DNA probe, a previously established protocol reported by the group of Plaxco has been reproduced [35]. Briefly, the anti-miR-224-DNA probe was reduced in the presence of 10 mM tris(2-carboxyethyl) phosphine hydrochloride (TCEP) for a duration of 1 h. TCEP plays crucial role in reducing the disulfide binding of the probe, preparing it for covalent bonding to AuNPs. Subsequently, the resulting solution was diluted to achieve the desired concentration in the nanomolar range, facilitating its immobilization on the AuNPs-SPE surface. The probe was immobilized on the working electrode area via drop casting 20 mL on the working electrode, following an incubation for 1 h in a humidity chamber at room temperature. The working electrode was gently washed with distilled water, and a solution of 2 mM of 6-mercapto-1-hexanol was placed on the working electrode area and incubated for 1.5 h in a humidity chamber washed with distilled water prior to use. If stored in buffer solution at 4 °C, the system allows a repeatable sensitivity up to 2 days.

### Measurement of miRNA target

Electrochemical measurements were conducted on eight electrodes simultaneously by inserting them into the eight-channel multiplexer potentiostat. Before measurement in the presence of target, the probe was allowed to stabilize in 100 mL of blank solution (PBS or untreated serum) for 30 min. Subsequently, miRNA target was added to the working solution, and the measurement was recorded after 30 min, to allow binding between the probe and miRNA. The signal was recorded using square wave voltammetry (SWV) as electrochemical technique with the following parameters: equilibrium time = 5 s, *E* start = 0 V, *E* end =  − 0.6 V, *E* step = 0.001 V, amplitude = 0.01 V, frequency = 50 Hz. Experimentally, in the presence of the target, a decrease in recorded current was observed due to the formation of a rigid duplex structure, which reduces electron transfer between MB and the electrode, yielding a signal off platform [22]. All the measurements have been performed in a controlled room temperature setting, comprised among 20–22 °C depending on temperature fluctuations during the day. The signal change (%) was calculated as follows:

$$Signal\;change\,\%=\left(i_\text{blank}-i_\text{target}\right)/i_\text{blank}\times100,$$

where *i*_blank_ and *i*_target_ are the currents recorded in absence and in presence of target, respectively.

### Preconcentration at paper-based disk

**Fig. 1:**
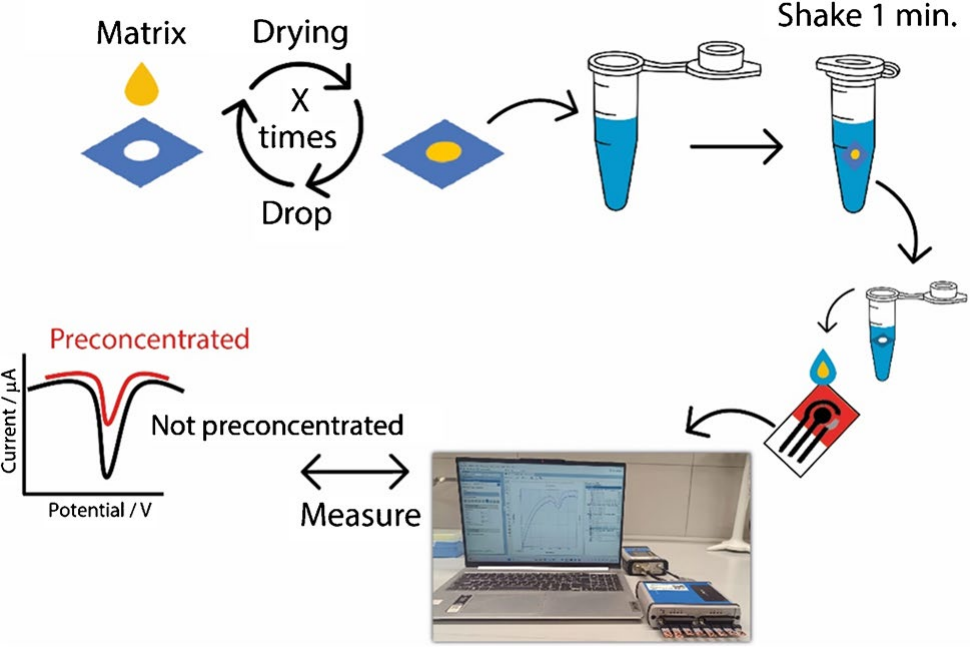
Schematic representation of the preconcentration protocol. A 2-mL droplet of solution containing the miRNA solution to be preconcentrated is placed on the disk via drop casting, followed by air drying. This procedure can be repeated *N* times. The disk is then transferred into a vial containing buffer solution and manually shaken for 1 min. Subsequently, the solution containing the preconcentrated target is measured via SWV at the paper-based electrode

A waxed Whatman No. 1 chromatographic paper was used to prepare the disk for preconcentration. The Whatman paper was chosen because it is renowned for its high porosity and reliability in the field of analytical devices as previously highlighted in literature [36, 37]. The configuration of the disks was designed using the software Adobe Illustrator, and the chosen diameter of the disk was equal to 3 mm. The disks were patterned with the use of the ColorQube 8580 wax printer which allowed to obtain a wax layer, subsequently thermically treated in the oven at 100 °C for 1 min, with the result of having a hydrophilic area surrounded by a three-dimensional hydrophobic barrier. As reported in Fig. 1, the preconcentration process was easily performed.

The miRNA target solutions to be preconcentrated, both in standard and human serum solutions, were added into the hydrophilic portion of the disk. A 2-mL drop of sample was added and the solvent (i.e., water) was allowed to evaporate at room temperature for ca. 1 min. The procedure was repeated for ten times, in order to have a 10 × preconcentration on the paper-based disk. After the preconcentration was concluded, the paper-based disk was inserted in a vial containing phosphate buffer. The vial tube was handily shaken for 1 min, and subsequently, the solution was placed on the electrochemical strip and analyzed. Along with all the measurements of preconcentrated solutions, the nominal non-preconcentrated targets were also measured to guarantee the effectiveness of procedure, e.g., if the 1 nM miRNA target was 10 × preconcentrated, the non-preconcentrated 10 nM miRNA was also measured.

## Results and discussions

### Optimization of the experimental parameters for miRNA detection

To develop the biosensor for miR-224 detection in serum, the most relevant experimental parameters were systematically optimized, namely the salts concentration, the amount of gold nanoparticles, the square wave frequency, and the amount of immobilized probe were taken into account. In particular, all the parameters investigated affect the sensitivity of the final platform; in fact, a high salt concentration might have an effect on the interaction between probe and target that are usually characterized by a high electrostatic repulsion due to the negatively charged backbones, crowded surface modified with recognizing probes might result in lower affinity with probe due to the difficulty of binding, the presence of AuNPs is necessary for attaching the recognizing probe but they might yield a thick surface which limits the conductivity, and the square wave frequency is essential to observe the signal variation during the electrochemical measurements [38]. As reported in the supporting information file, Fig. S1, a concentration of 140 mM sodium chloride, a square wave frequency of 50 Hz, 4 µL of AuNPs, and a 50 nM of starting concentration of probe were immobilized. These conditions represented the optimal compromise in terms of sensitivity, repeatability, mimicking physiological conditions, and low cost (associated with the cost of reagents).

### Analytical performances and selectivity of the electrochemical strip

**Fig. 2:**
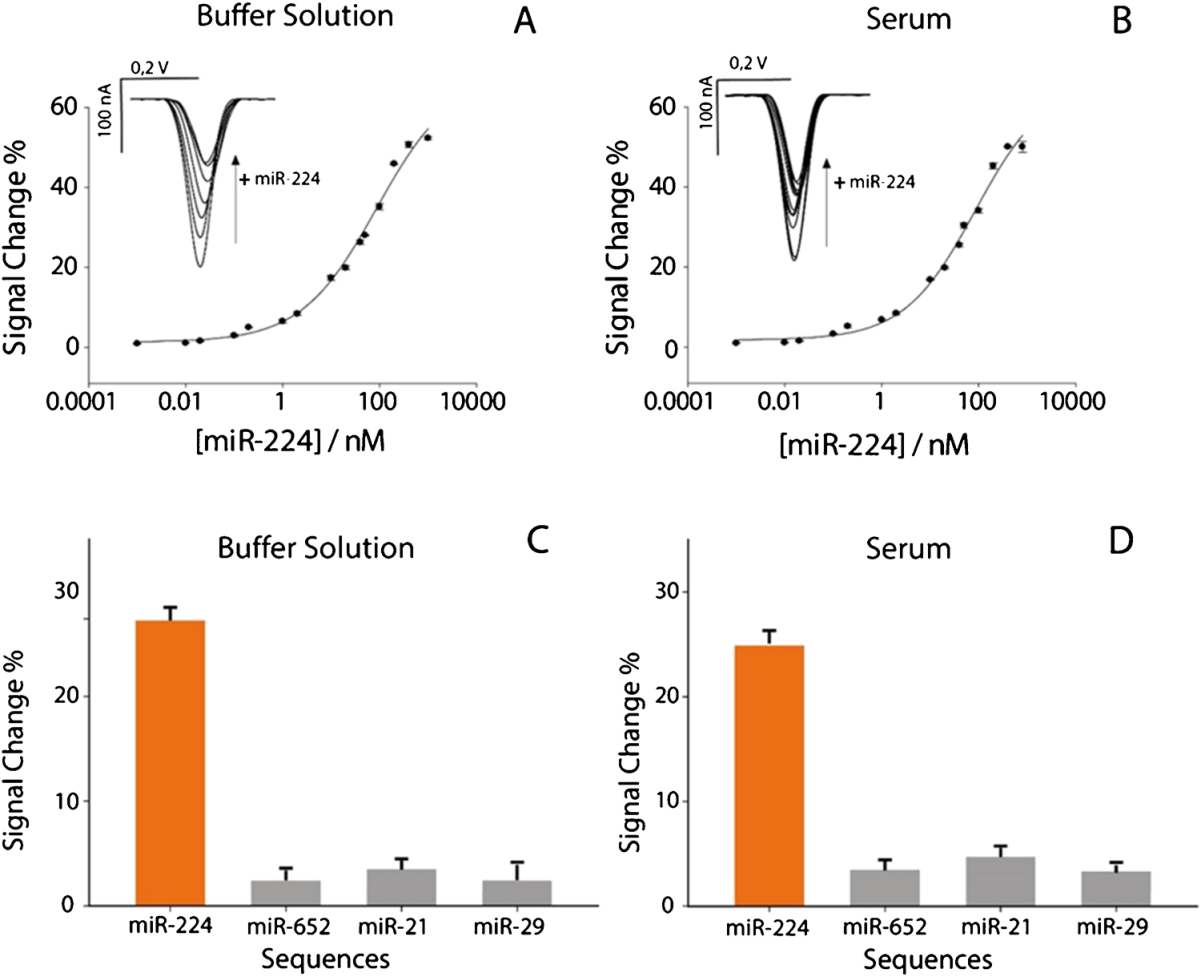
**A** Calibration curve and SWV curves obtained in buffer solution by testing different concentrations of miR-224 target from 0.01 to 800 nM. **B** Calibration curve and SWV curves obtained in human serum by testing different concentrations of miR-224 from 0.01 to 800 nM. All the experiments have been carried out in triplicate. SWV parameters were as follows: *t* eq = 5 s, *E* start = 0.0 V, *E* end =  − 0.6 V, *E* step = 0.001 V, amplitude = 0.01 V, frequency = 50.0 Hz. Selectivity studies comparing signal intensities obtained in the presence of 50 nM miR-224 and in the presence of noncomplementary miRNA single strands, in **C** buffer solution and **D** human serum

The analytical performance of the biosensor was evaluated in phosphate buffer solution and in commercial human serum. Working solutions were spiked with levels of miR-224 ranging from 1 pM to 800 nM, using the optimized experimental parameters discussed in the previous paragraph. As can be seen in Fig. 2, a characteristic semi-logarithmic sigmoidal correlation emerged between the signal change % and the logarithmic scale of target concentration expressed in nanomolar.

Intriguingly, this sigmoidal trend was evident in both buffer solution and whole serum measurements, confirming the robustness and versatility of the method in different biological sample matrices. High correlations of 0.99 were observed for miR-224 targets in both solutions. In addition, the limit of detection, calculated at the concentration corresponding to a 10% signal change, was found to be ca. 0.6 nM in both buffer solution and human serum. The serum matrix therefore does not hinder the monitoring of miR-224. The repeatability of the platform was evaluated, calculated as relative standard deviation (RSD%). The paper-based strips showed a RSD% lower than 5%, confirming the promising results and providing advantages in simplicity, production efficiency, sensitivity, and cost-effectiveness. The selectivity of the platform was evaluated in the presence of three random interfering oligonucleotides, namely miR-652 (5′-caa ccc uag gag ggg gug cca ucc-3′), miR-21 (5′-uag cuu auc aga cous agu uga-3′), and miR-29a (5′-tag cac cat ctg aaa tcg gtt-3′), as reported in Fig. 2. The tests were conducted both in buffer solution and in human serum spiked with 50 nM miRNA. In all cases, a nonsignificant signal change, less than 5%, was detected. This result unequivocally highlights the high selectivity of the detection platform, confirming its effectiveness in specifically identifying the target of interest.

### Target preconcentration on filter paper disks

**Fig. 3:**
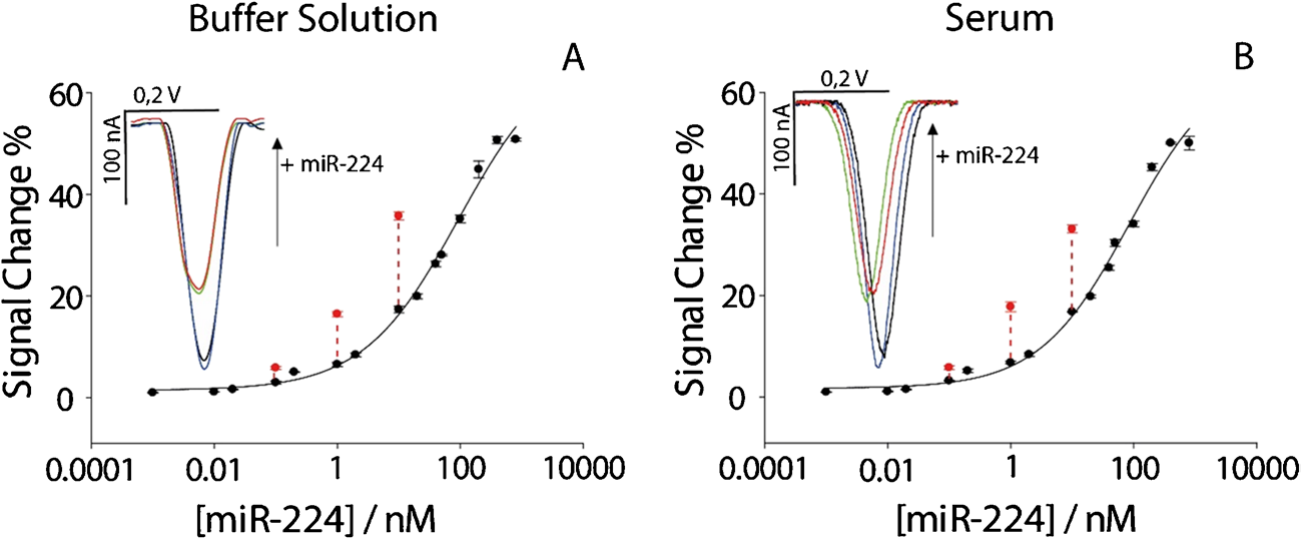
Calibration curves showcasing the effects of preconcentration on target detection. **A** Calibration curve in buffer solution. **B** Calibration curve in human serum. All the experiments have been carried out in triplicate. Insets display voltammograms illustrating target presence and concentration effects: blue curve indicates the absence of target, black curve represents the presence of a 1 nM target, red curve shows a 1 nM target preconcentrated 10 times, and green curve denotes the presence of a 10 nM target

Although the reported approach has been characterized by satisfactory analytical performance, what should be considered is the possibility to improve the performance of analytical methods which are characterized by some physical limitations. In fact, even if the use of nanomaterials might be applied towards the detection of nucleic acids with the goal of obtaining high sensitivity, what should be noted is that the sensitivity of hybridization-based devices is strongly dependent on the affinity between probe and target. It means the binding event is the major limiting feature in defining the sensitivity of these approaches. As described above, the use of preconcentration techniques like magnetic beads, enzyme amplification, solvent evaporation, and PCR has the role of increasing the effective concentration to be tested, thus resulting in better outcomes in terms of detection. These approaches might be time consuming and costly, both considering the reagents, equipment, and personnel. Instead, what is characterized and presented here is the adoption of chromatographic paper disk acting as a frugal preconcentration. The advantages associated with the use of porous paper are well-known: its porosity guarantees a high pre-loading of reagents, and it offers the possibility to work with diverse matrices, including human serum. Herein, the focus was on the significant contribution of a waxed paper-based disk for detection of lower concentration of miRNA without affecting the complexity of the whole architecture, taking as model case the detection of miR-224. To evaluate the capacity of paper-based disk to act as a frugal preconcentration tool, 0.1, 1, and 10 nM of miR-224 were interrogated. Briefly, 2 mL of the chosen concentration of miR-224 was drop cast for ten times onto a paper-based disk, and each addition was carried out after the solvent from the previous drop evaporated. Successively, the preconcentrated paper-based disk was immersed into a vial preloaded with 50 mL of buffer solution to release the adsorbed miRNA. After shaking the disk for a minute in the buffer solution, 50 mL was measured onto the office paper-based electrochemical strip, obtaining satisfactory results as reported in Fig. 3.

**Fig. 4:**
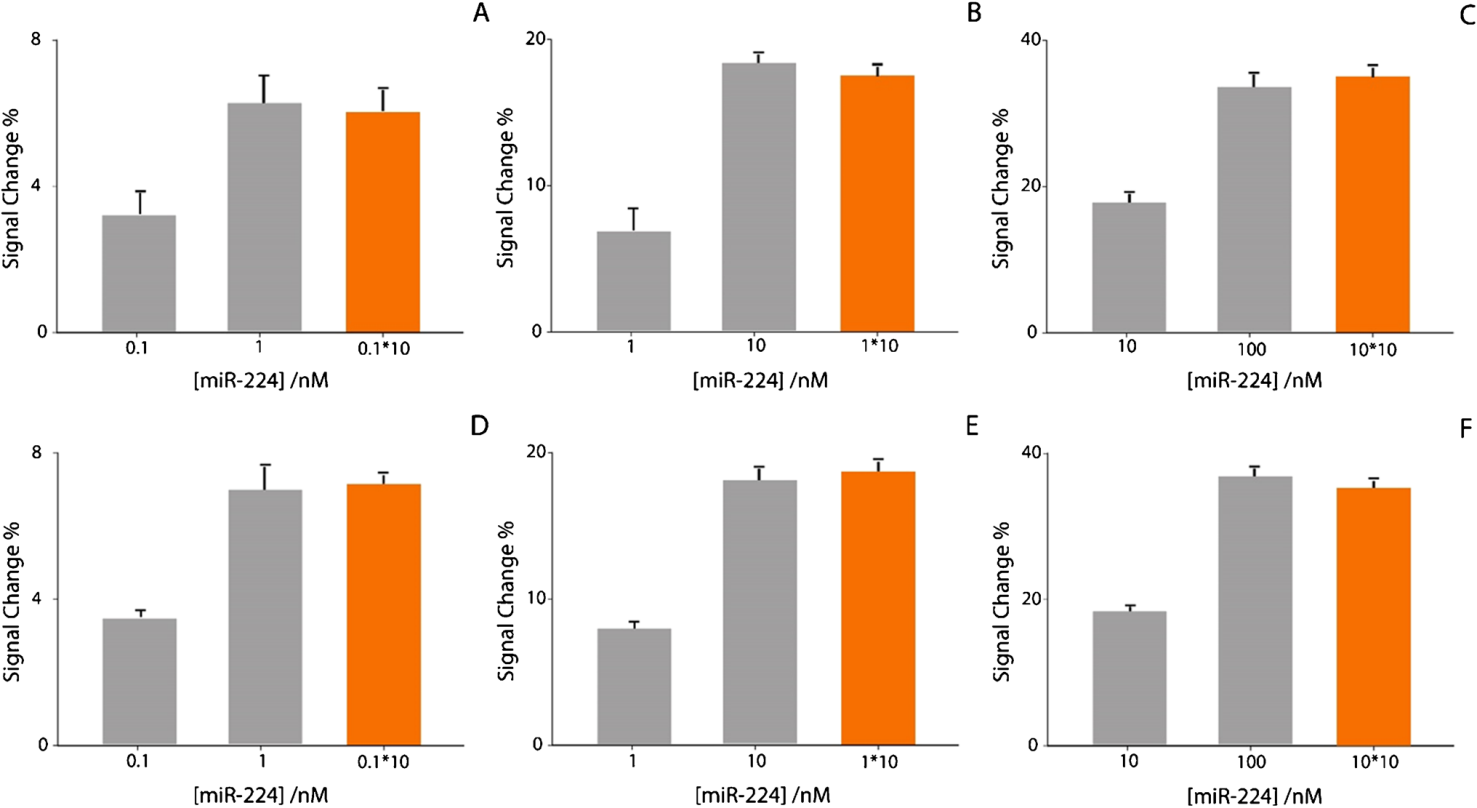
Preconcentration study comparing different concentrations of preconcentrated target (in orange) and non-preconcentrated target (in gray). **A** 0.1 nM in buffer solution. **B** 1 nM in buffer solution. **C** 10 nM in buffer solution. **D** 0.1 nM in human serum. **E** 1 nM in human serum. **F** 10 nM in human serum. All the experiments have been carried out in triplicate

The preconcentration process resulted in an increase of the signal change, for both buffer and human serum solutions. In particular, the three levels of miR-224, namely 0.1, 1, and 10 nM, were taken as examples for demonstrating this effective approach. In particular, to evaluate the efficacy, all the signal changes due to preconcentrated levels of miRNA have been compared with the non-preconcentrated solutions, as reported in histograms in Fig. 4.

A preconcentration of tenfold was reflected in an improvement of signal change of tenfold, confirming the effectiveness of the approach. A note of merit of the paper-based disk is the possibility to be applied towards human serum without pretreatments, and the lowering of the detection of miR-224 down to 50 pM, with only ca. 10 min more with respect to the non-preconcentrated system.

## Conclusion

In this work, a new and frugal approach has been adopted to preconcentrate miRNA in biological samples. The idea of using a paper-based disk to concentrate miR-224 is very novel as it involves combining chromatographic paper with a printed electrochemical strip. This method takes advantage of the nature and adsorption capabilities of the paper, enabling preconcentration of selected compounds. By utilizing the paper-based matrix to concentrate compounds through adsorption and solvent evaporation, the sensitivity was effectively enhanced. Thanks to intrinsic features of porous chromatographic Whatman No. 1 paper, it was possible to amplify the concentration of target miRNA with the use of a very simple procedure, just using a paper-based disk that was previously waxed to define the preconcentration area. In fact, a 3-mm-diameter chromatographic paper-based disk was used as the support where the waxed preconcentration area was printed and it was possible to be loaded with multiple aliquots of target. The designed area was effective in hosting 2 µL of sample without a significant coffee ring effect on the edges of the disk, and the efficacy of the method was consistent with the possibility of programming the number of preconcentration steps, just adding 2 µL more. In order to demonstrate the efficacy of the concept, an office paper-based electrochemical device for detecting miR-224, associated with lung cancer prognosis, has been taken into account. Considering the detection limit of the device calculated in both buffer and human serum, equal to ca. 0.6 nM, it should be noted that the application of ten steps of preconcentration on the paper-based disk led to a ca. 50 pM of calculated detection limit. What should be highlighted is the absence of time-consuming and complex procedures; in fact, only a paper-based disk, a vial, and 10 min are required to perform the preconcentration. The preconcentration efficacy was evaluated and characterized at three levels of miR-224, demonstrating a satisfactory correlation among results. In general, the use of paper-based substrates is not only capable to provide a sustainable substrate for manufacturing sensors and biosensors, but it offers the possibility to enhance the analytical performance of plenty of methods. It represents a generalizable approach that might be applied to various transduction architectures, electrochemical, optical, plasmonic, etc., and also to different matrices. The exploitation of paper-based architectures is suitable for the development and the enhancement of portable and decentralized possibilities, for all the field of application, reducing the necessity of costly procedures and specialized personnel.

### Supplementary Information

Below is the link to the electronic supplementary material.

Supplementary file1 (DOCX 91 KB)
