# Supplementary material for: Enhancing sensitivity towards electrochemical miRNA detection using an affordable paper-based strategy
Source: Anal Bioanal Chem. 2024 Jun 20;416(19):4227–36. doi: 10.1007/s00216-024-05406-6 (PMC11271339; doi:10.1007/s00216-024-05406-6)
Supplement: Supplementary file 1 — Supplementary file1 (DOCX 91 KB) [file 216_2024_5406_MOESM1_ESM.docx]

Supplementary information

**Enhancing sensitivity towards electrochemical miRNA detection using an affordable paper-based strategy**

Wanda Cimmino^1^, Ada Raucci^1^, Sara Pia Grosso^1^, Nicola Normanno^2^, Stefano Cinti^1*^

^1^Department of Pharmacy, University of Naples “Federico II”, Via Domenico Montesano 49, 80131 Naples, Italy.

^2^ Cell Biology and Biotherapy Unit INT-Fondazione Pascale, Naples, Italy

*Corresponding author: Stefano Cinti [Stefano.cinti@unina.it](mailto:Stefano.cinti@unina.it)

Optimization of the experimental parameters for miRNA detection


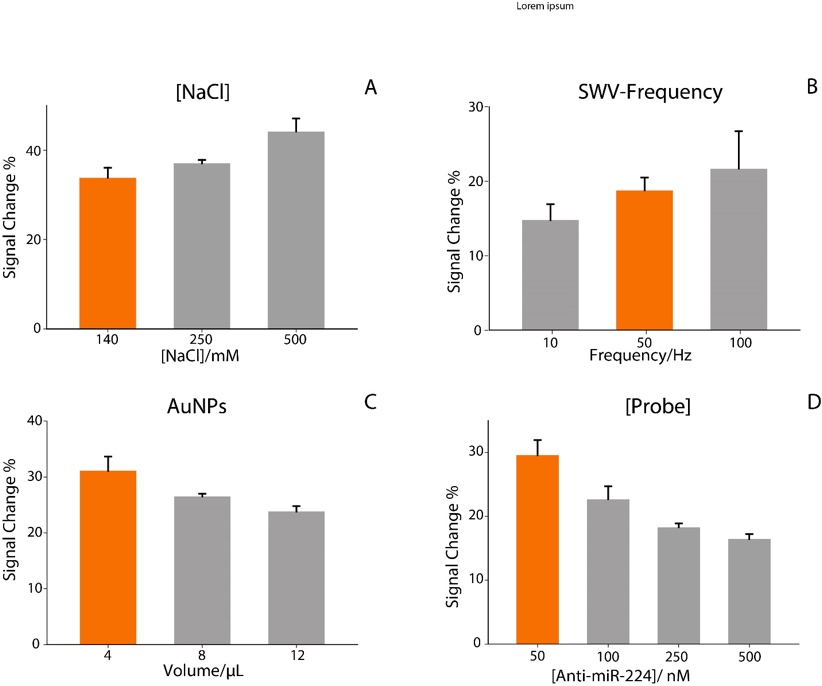


**Figure S1** A) Study of the concentration of sodium chloride in the working buffer solution. B) Study of the square-wave frequency between 10 and 100. C) Study of the volume of AuNPs with which to change the surface area of the electrode working area D) Study of anti-miR-224 specific probe concetration at 50, 100, 250 and 500 nM. All the optimization was carried out in presence of a target concentration of 40 nM. The orange bars represent the optimized value
